# Intestinal Malrotation in Adults: A Retrospective Cross-Sectional Study Based on Computed Tomography Findings

**DOI:** 10.7759/cureus.114908

**Published:** 2026-08-21

**Authors:** Natalia Denisiewicz, Miłosz Korbaś, Dominika Kaczyńska, Adam Mitręga, Zofia Mandrela, Jakub Kufel

**Affiliations:** 1 Department of Radiology and Nuclear Medicine, Medical University of Silesia, Katowice, POL; 2 Department of Biophysics, Medical University of Silesia, Katowice, POL; 3 Department of Radiodiagnostics and Radiology, Professor Kornel Gibiński University Clinical Center, Katowice, POL; 4 Department of Radiology and Nuclear Medicine and Department of Radiodiagnostics and Invasive Radiology, Medical University of Silesia, Katowice, POL

**Keywords:** abdominal imaging, adult population, computed tomography, congenital anomalies, intestinal malrotation

## Abstract

Purpose

Intestinal malrotation is an uncommon congenital anomaly that may remain clinically silent until adulthood and is increasingly detected incidentally on computed tomography (CT). Classical radiologic markers have limited diagnostic sensitivity in adults. This study evaluated the prevalence and CT characteristics of intestinal malrotation in a large adult cohort using strict CT-based diagnostic criteria.

Methods

A retrospective cross-sectional review of 3,478 abdominal CT scans performed between June 2023 and April 2025 was conducted. All examinations were screened for features suggestive of rotational anomalies. Suspected cases underwent a two-stage verification process by two independent radiologists. Classification as intestinal malrotation required assessment of the predominant jejunal location, cecal position, and duodenal course. Prevalence estimates and proportions were calculated with Wilson 95% confidence intervals (CI).

Results

Intestinal malrotation was identified in seven of 3,478 CT examinations (0.20%; 95% CI: 0.10%-0.42%). Two additional cases demonstrated atypical bowel positioning but did not fulfill the predefined primary diagnostic criteria and were excluded. The mean age of the confirmed cases was 64 ± 14 years. Gastrointestinal alarm symptoms occurred in 28.6% (two of seven) of patients, while most cases were asymptomatic. One patient presented with fatal intestinal obstruction.

Conclusions

Adult intestinal malrotation was uncommon and frequently detected incidentally in this CT cohort. Systematic assessment of multiple anatomical parameters may improve diagnostic confidence, as isolated anatomical deviations may mimic malrotation. These findings should be interpreted cautiously because the diagnoses were based exclusively on CT criteria, and the number of confirmed cases was small.

## Introduction

Intestinal malrotation is a congenital defect resulting from abnormal rotation and fixation of the midgut around the axis of the superior mesenteric artery (SMA) during fetal development. Under normal circumstances, the midgut undergoes a 270° counterclockwise rotation during embryogenesis. Any deviation from this process is termed malrotation. Several forms of this anomaly are recognized within a clinical spectrum, including nonrotation, reversed rotation, and incomplete rotation [1]. Complete nonrotation is the most common and clinically significant form because the small bowel remains predominantly on the right side of the abdomen and the colon on the left, resulting in an abnormally narrow mesenteric base [2]. This anatomical configuration increases the risk of midgut volvulus and bowel obstruction. Abnormal intestinal fixation may also result in Ladd’s bands, which can contribute to bowel obstruction [3-5]. Evidence suggests that the key pathophysiological factor responsible for complications in malrotation is not the abnormal positioning of intestinal loops itself, but rather defects in mesenteric fixation. It is this lack of anatomical stabilization, rather than the relative location of the bowel, that plays a central role in the development of clinically significant complications [2].

Intestinal malrotation is typically recognized as a pediatric condition, as its life-threatening complications most often present within the first month of life. In adults, this diagnosis is rare and usually discovered incidentally during imaging studies performed for other reasons. The incidence of malrotation in newborns is estimated at one in 500 live births [6], although this number may be underestimated since some cases remain asymptomatic throughout life. In adults, the true prevalence is difficult to determine due to numerous undiagnosed cases; however, it is estimated to be approximately 0.2%-0.5% in the general population [4,5].

Contrast agents play a key role in the diagnosis of intestinal malrotation, enabling detailed assessment of bowel positioning and their relationship to anatomical structures. The gold standard, particularly in the pediatric population, remains the upper gastrointestinal (UGI) series with oral contrast administration, which allows visualization of the abnormal course of the duodenum and the absence of the typical duodenal loop at the level of the ligament of Treitz [7]. In adults, computed tomography (CT) with intravenous iodinated contrast is gaining increasing importance [8]. These modalities allow evaluation of bowel malposition, mesenteric root width, the presence of adhesions, and possible signs of volvulus (e.g., whirlpool sign and retromesenteric duodenum) [9-11]. In certain cases, especially during CT colonography (CTC), rectal contrast agents such as air or water-soluble iodine solutions are also used. These can help visualize the abnormal positioning of the colon and the mesenteric vessels, even in asymptomatic individuals [2]. The choice of contrast type depends on the patient’s age, clinical presentation, and the availability of diagnostic methods, and its proper use is crucial for diagnosing this potentially life-threatening congenital anomaly.

In adult patients, intestinal malrotation may be minimally symptomatic or present with non-specific, chronic complaints, which contributes to frequent diagnostic challenges. In cases of incomplete malrotation, symptoms may be entirely absent. Chronic manifestations may include recurrent abdominal pain, bloating, nausea, food intolerance, intermittent vomiting, and weight loss [12]. In many instances, these symptoms are mistakenly attributed to other functional gastrointestinal disorders. In the acute form, most commonly associated with intestinal volvulus, symptoms have a sudden onset and include severe abdominal pain, nausea, vomiting, and signs of bowel obstruction or ischemia. Such conditions can lead to shock and require urgent surgical intervention. Due to the variability and non-specific nature of the clinical presentation, malrotation should be considered in the differential diagnosis of adult patients with unexplained abdominal complaints, particularly if they are recurrent and unresponsive to standard treatment [3].

Management following the detection of intestinal malrotation depends on the presence of clinical symptoms and the patient’s overall condition. In symptomatic cases, especially those involving acute volvulus or obstruction, emergency surgery is required, with the Ladd procedure being the standard approach [3]. This procedure involves division of Ladd’s fibrous bands, widening of the mesenteric base, repositioning of the intestines with the small bowel on the right and the colon on the left, and typically prophylactic appendectomy [13]. In patients with chronic, non-specific symptoms such as recurrent abdominal pain, nausea, or bloating, surgical treatment may also be considered, particularly when symptoms are persistent and impair quality of life. There is ongoing debate in asymptomatic cases diagnosed incidentally, as no clear guidelines exist regarding the need for surgery. Some studies recommend observation only [14], while others advocate for prophylactic surgical intervention due to the potential risk of sudden complications [4,15]. Therefore, treatment decisions must be made on an individual basis, taking into account not only the clinical presentation but also the potential risk of future complications.

The diagnosis of intestinal malrotation in adults remains a significant clinical challenge, despite the increasing availability of cross-sectional imaging. There is no single, definitive criterion that allows for a conclusive confirmation of this developmental anomaly in the adult population. The presence of isolated or so-called ancillary features, such as a reversed SMA-SMV (superior mesenteric vein) relationship or displacement of small-bowel loops, may only suggest the possibility of malrotation but does not provide a basis for a definitive diagnosis without simultaneous assessment of key anatomic parameters. Only a comprehensive analysis of the duodenal course and the predominant location of the jejunum and cecum enables reliable classification of rotational abnormalities. The complexity of the imaging findings and the frequent overlap with normal anatomic variants underscore that the diagnosis of malrotation in adults requires particular caution and the use of consistent, multi-parameter assessment criteria [1,8,16].

Therefore, the primary objective of this retrospective cross-sectional study was to estimate the prevalence of adult intestinal malrotation using strict, CT-based diagnostic criteria. Secondary objectives were to characterize its multi-parameter imaging spectrum, differentiate true malrotation from isolated anatomical variants, and describe associated clinical presentations.

## Materials and methods

The study was conducted retrospectively based on the analysis of abdominal and pelvic CT examinations performed between June 2023 and April 2025. A total of 3478 CT scans obtained for various clinical indications were reviewed, representing a cross-sectional analysis of a cohort of consecutive CT scans. Inclusion criteria encompassed examinations performed using a standardized protocol that permitted complete assessment of the gastrointestinal tract for features of abnormal intestinal rotation. Exclusion criteria included CT scans of anatomical regions other than the abdomen and pelvis, and examinations performed prior to June 2023. Although poor image quality or incomplete field of view were predefined as exclusion criteria (Appendix 1), ultimately zero patients were excluded for this reason. In routine practice, patients with non-diagnostic scans underwent repeat imaging, and their subsequent diagnostic-quality scans were included in the analysis.

All 3478 CT examinations were performed using a Siemens SOMATOM Definition Edge scanner (Siemens Healthineers AG, Erlangen, Germany) with a spiral, multiphase acquisition protocol. Standardized intravenous iodinated contrast (Omnipaque 350) was administered at a weight-based dose of 1.2 mL/kg via an automatic power injector, with injection duration adjusted according to total volume and patient body habitus. Imaging routinely included non-contrast, arterial, and portal venous phases, with an additional delayed/excretory phase acquired three to five minutes later when clinically indicated (e.g., for genitourinary or oncological evaluation). Images were reconstructed at slice thicknesses of 0.75 mm and 1.5 mm, and multiplanar reconstructions (axial, coronal, and sagittal planes) were systematically generated. Oral contrast preparation consisted of a 3% aqueous solution prepared by diluting Omnipaque in 1.5 L of water, which was administered to the patient two hours prior to the CT examination, provided that renal function parameters met established safety criteria.

Regarding image analysis, the purpose was to identify anatomical features consistent with intestinal malrotation and to classify them according to established CT-based criteria using multiplanar reconstructions in axial, coronal, and sagittal planes. The assessment was conducted in a two-stage process. Initially, all 3478 examinations were independently screened in a two-stage workflow involving radiology residents and board-certified radiologists with over 10 years of experience in abdominal imaging (Z.M. and J.K.). The readers were blinded to the original CT reports, patient clinical indications, and each other’s assessments. At this stage, the assessment focused on the presence of imaging signs, including a predominance of jejunal loops on the right side, an atypical course of the duodenum, a suspected absence of the retromesenteric D3 segment, an atypically positioned cecum or ileocecal valve (ICV), as well as the presence of a whirlpool-like configuration of mesenteric vessels. Any discrepancies regarding the presence of these features were systematically recorded during the retrospective review process, and any examination flagged as potentially consistent with malrotation by either reader was advanced to the second stage for detailed verification.

Subsequently, all candidate cases underwent a definitive evaluation using the formal classification system proposed by Xiong et al. [8]. The Xiong classification was chosen because it offers a structured, multiparametric CT-based assessment specifically applicable to adult intestinal malrotation. This system categorizes intestinal malrotation in adults according to three key features evaluated on CT images: (i) the course of the duodenum, specifically whether it crosses the midline between the aorta and the SMA; (ii) the predominant location of the jejunum; and (iii) the position of the cecum. Crossing of the midline was defined as any passage of the horizontal duodenal segment from right to left across the sagittal midline, regardless of its relationship to the mesenteric vessels. For the purpose of this study, a retromesenteric D3 segment was defined as a horizontal duodenal segment coursing posterior to the superior mesenteric vessels, within the retroperitoneum. Absence of a retromesenteric D3 was recorded when the horizontal duodenum did not follow this course, including anterior passage relative to the mesenteric vessels or inability to demonstrate a typical retromesenteric trajectory within the assessed CT volume. Crucially, while a normal retromesenteric D3 course is typical, adult malrotation presents heterogeneously. To address borderline cases and prevent underdiagnosis, we adopted a consensus-based definition whereby a preserved D3 did not strictly exclude the diagnosis if concurrent, unequivocal abnormalities in jejunal and cecal positioning were identified. The minimum required combination of findings to define “true intestinal malrotation” was the concurrent presence of an abnormal predominant jejunal location and an aberrant cecal position, verified by consensus. Based on these parameters, the authors defined ten subtypes of malrotation reflecting varying degrees of abnormal midgut fixation. Additional anatomical parameters were also systematically assessed to support the diagnosis. The position of the ICV was categorized into one of four quadrants: right lower (RLQ), right upper (RUQ), left lower (LLQ), or left upper (LUQ). The SMA-SMV relationship was classified as normal (SMA to the left of SMV), inverted (SMA to the right), or vertical (SMA posterior to SMV). Furthermore, the location of the ligament of Treitz was analyzed to determine whether it was situated in its typical position on the left side near the duodenojejunal junction, and any prominent or spiral configuration of mesenteric veins (“whirlpool sign”) was recorded.

Final classification was established by mutual agreement in a joint consensus session between the two primary readers. In cases of ongoing diagnostic disagreement, a third independent senior abdominal radiologist served as an adjudicator, reviewing the images and findings against the established Xiong criteria to make the final binding decision. Only examinations fulfilling the strict criteria for true intestinal malrotation were included in the final study group, whereas cases presenting with isolated variants were excluded. The final diagnoses were based exclusively on the predefined CT criteria, as no additional imaging or surgical reference standard was available. Statistical analysis included descriptive statistics (mean ± SD, percentages) and the calculation of 95% confidence intervals (CIs) using the Wilson score interval method. Interobserver agreement for the initial screening stage was quantified using Cohen’s kappa coefficient (κ). The reporting of this study conforms to the STROBE guidelines [17] for cross-sectional studies (Appendix 2).

## Results

Out of the 3478 examinations reviewed, the initial independent screening yielded a total of nine cases flagged as suspicious for rotational abnormalities by at least one reader. Reader 1 identified all nine suspected cases, whereas Reader 2 identified seven of these cases. As there were no cases identified by Reader 2 that were missed by Reader 1, the interobserver agreement for the initial screening was high (Cohen’s κ = 0.87). The raw agreement counts were seven positive agreements, two disagreements, and 3469 negative agreements. Subsequently, all nine candidate cases underwent a second-stage definitive evaluation using the formal classification system proposed by Xiong et al. [8]. During this detailed verification, two cases, referred to as patients 8 and 9, were found to demonstrate only atypical bowel positioning. Crucially, in these two patients, the three primary criteria of the Xiong classification - the position of the duodenum, the predominant location of the jejunum, and the position of the cecum - showed no deviations from normal anatomy. Since the observed abnormalities involved solely supplementary parameters without fulfilling the primary definition, these two cases were excluded. Consequently, the final study group consisted of seven confirmed cases of intestinal malrotation, yielding an overall prevalence of 0.20% (95% CI: 0.10%-0.42%).

The confirmed study group comprised seven patients (four females and three males) with a mean age of 64 ± 14 years. The seven patients underwent CT for various, potentially overlapping clinical indications, which are summarized in Table 1. Because individual patients could have more than one indication, the total number of indications exceeded the number of confirmed cases.

**Table 1:** Indications for CT examination among patients with confirmed intestinal malrotation. CT: computed tomography

Indication for CT examination	Number of patients
Acute Abdominal Pathology	3
Oncological Indication	3
Monitoring Focal/Chronic Pathology	3

A history of previous abdominal surgery was noted in four patients. Regarding clinical presentation, gastrointestinal alarm symptoms extracted retrospectively from CT referral forms, such as abdominal pain, weight loss, prolonged diarrhea, or diarrhea with blood, were reported in two out of seven patients (28.6%; 95% CI: 8.2%-64.1%). Chronic nonspecific symptoms were not systematically evaluated. However, it should be noted that the absence of these symptoms does not rule out the presence of intestinal malrotation, as the majority of the cohort was asymptomatic. In terms of outcomes, features of intestinal malrotation were identified in one case on an emergency CT scan performed due to symptoms of gastrointestinal obstruction and advanced malnutrition. Despite intensive treatment, this patient died. While imaging strongly suggested complications from impaired intestinal transit, this finding lacks surgical or autopsy confirmation. In the remaining cases, there were no signs of acute intestinal ischemia or advanced obstruction. A review of medical records indicated that these patients were not referred for urgent surgical consultation, nor did clinical management plans include elective surgery solely for the diagnosed malrotation.

Detailed anatomical indicators were assessed to differentiate true malrotation from variants. Table 2 presents a comparison of gastrointestinal structure locations between the confirmed malrotation group (n=7) and the excluded atypical cases (n=2).

**Table 2:** Location of gastrointestinal structures and detected anomalies in nine patients suspected of having intestinal malrotation. SMA: superior mesenteric artery; SMV: superior mesenteric vein; ICV: ileocecal valve; RLQ: right lower quadrant; RUQ: right upper quadrant; LLQ: left lower quadrant; LUQ: left upper quadrant

Examined parameter	Variant	Suspected cases - number of patients	Confirmed cases - number of patients
Position of the duodenum	Duodenal course crossing the midline	7	5
				Does not cross midline	2	2
	Retromesenteric horizontal duodenum (D3)	Present	7	5
Absent		2	2
Predominant location of the jejunum and ileum	Entirely on the right side	5	5
	In the right upper quadrant	1	1
	Initial part on the right, distal segments on the left	3	1
				Position of ileocecal valve (ICV)	RUQ	-	-
LUQ	-	-		
RLQ	7	5		
LLQ	1	1		
Midline	1	1		
SMA-SMV relationship	Normal	8	6
	Abnormal	1	1
Presence of the “whirlpool sign”	Present	4	2
	Absent	5	5
Position of the ligament of Treitz	Atypical - on the right side	1	1
	Normal	8	6
Other associated abnormalities	Pancreatic cyst/tumor, splenic cyst, accessory spleen, dilated bowel loops, colonic diverticula	6	5
	Absent	3	2

A distinct anatomical divergence was confirmed: while the seven confirmed cases exhibited consistent abnormalities in jejunal and cecal positioning, the two excluded cases demonstrated normal anatomy regarding these primary parameters. Notably, regarding duodenal anatomy, absence of a retromesenteric D3 segment was identified in only two out of seven confirmed patients, despite midline crossing of the duodenum being present in the majority of cases. The remaining five confirmed cases demonstrated a preserved retromesenteric D3 course despite significant rotational anomalies of the midgut. Among the confirmed group, intestinal malrotation was classified according to the specific subtypes defined by Xiong et al. [8]. Individual demographic characteristics, clinical presentation or indication for CT, and malrotation subtype for each confirmed case are summarized in Table 3.

**Table 3:** Demographic characteristics, clinical presentation or indication for CT, and malrotation subtype according to the Xiong classification in patients with confirmed intestinal malrotation. Detailed Xiong system abbreviations: 1st letter = Duodenum (D); 2nd letter = relationship to midline (Y = crosses midline (Yes), N = does not cross (No), P = partial); 3rd letter = Jejunum (J); 4th letter = predominant jejunal position (R = Right, L = Left); 5th letter = Cecum (C); 6th letter = cecal position (R = Right, L = Left, M = Middle, P = Pelvic). CT: computed tomography

Patient	Age (years)	Sex	Clinical presentation/indication for CT	Type of malrotation
1	42	Male	Diagnostic work-up for sarcoidosis	DYJRCL
2	40	Male	Follow-up and evaluation of a pancreatic tail cyst; oncological indication	DYJRCR
3	68	Female	Surveillance of a complex cystic lesion in the abdominal cavity; oncological indication	DYJRCR
4	70	Female	Assessment of intestinal anastomotic stenosis in the course of chronic Crohn’s disease	DYJRCM
5	62	Female	Oncological indication	DNJRCR
6	79	Male	Abdominal pain and diarrhoea	DYJLCR
7	70	Female	Assessment of colonic diverticula	DNJRCR

**Figure 1:**
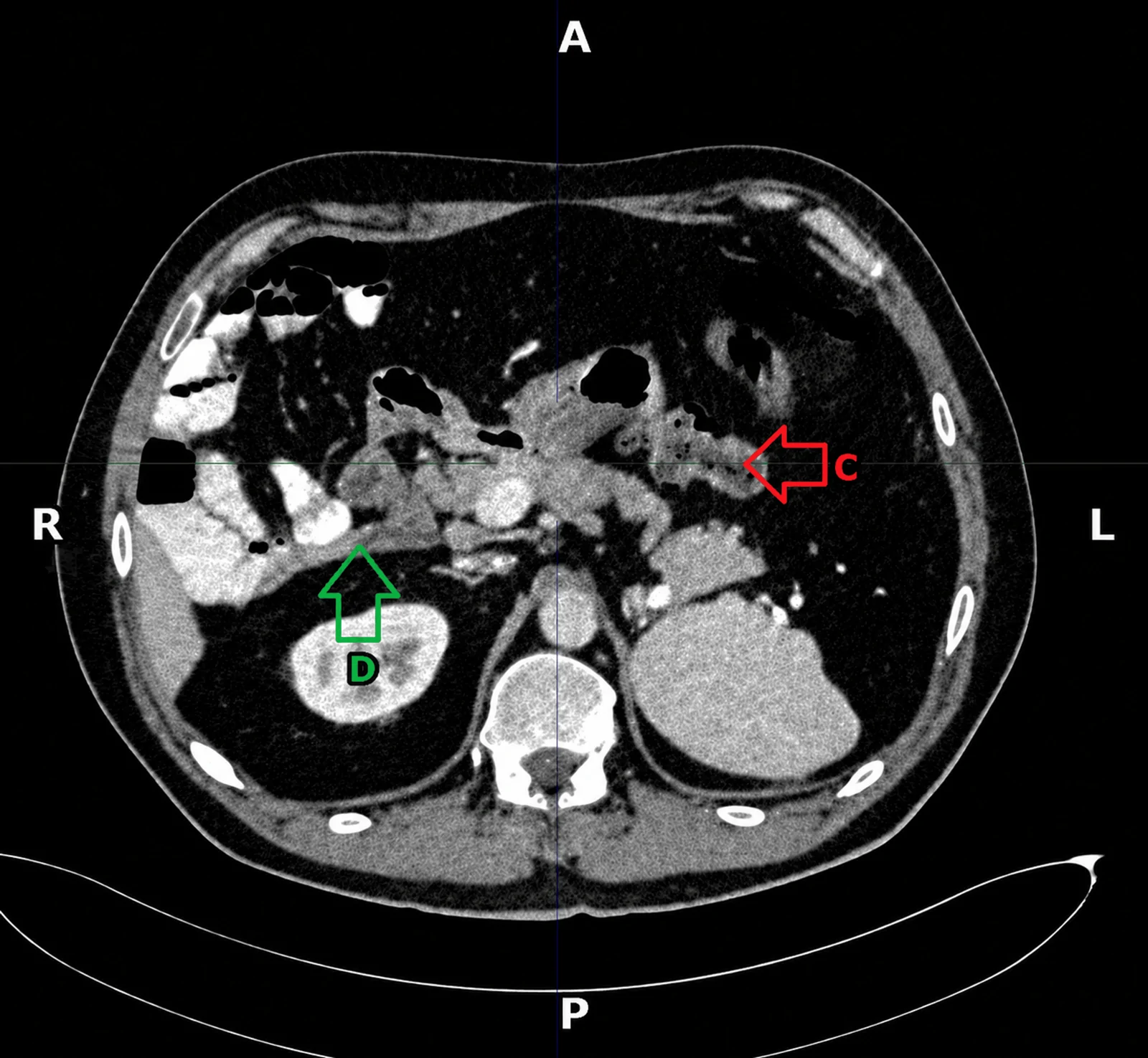
Axial contrast-enhanced CT image of patient 1 demonstrating right-sided predominance of the duodenum and jejunal loops, a midline course of the ascending colon, and a predominantly left-sided location of the remaining colon. The green arrow labeled D indicates the duodenum, and the red arrow labeled C indicates the colon. R, L, A, and P indicate the patient’s right, left, anterior, and posterior sides, respectively. CT: computed tomography

**Figure 2:**
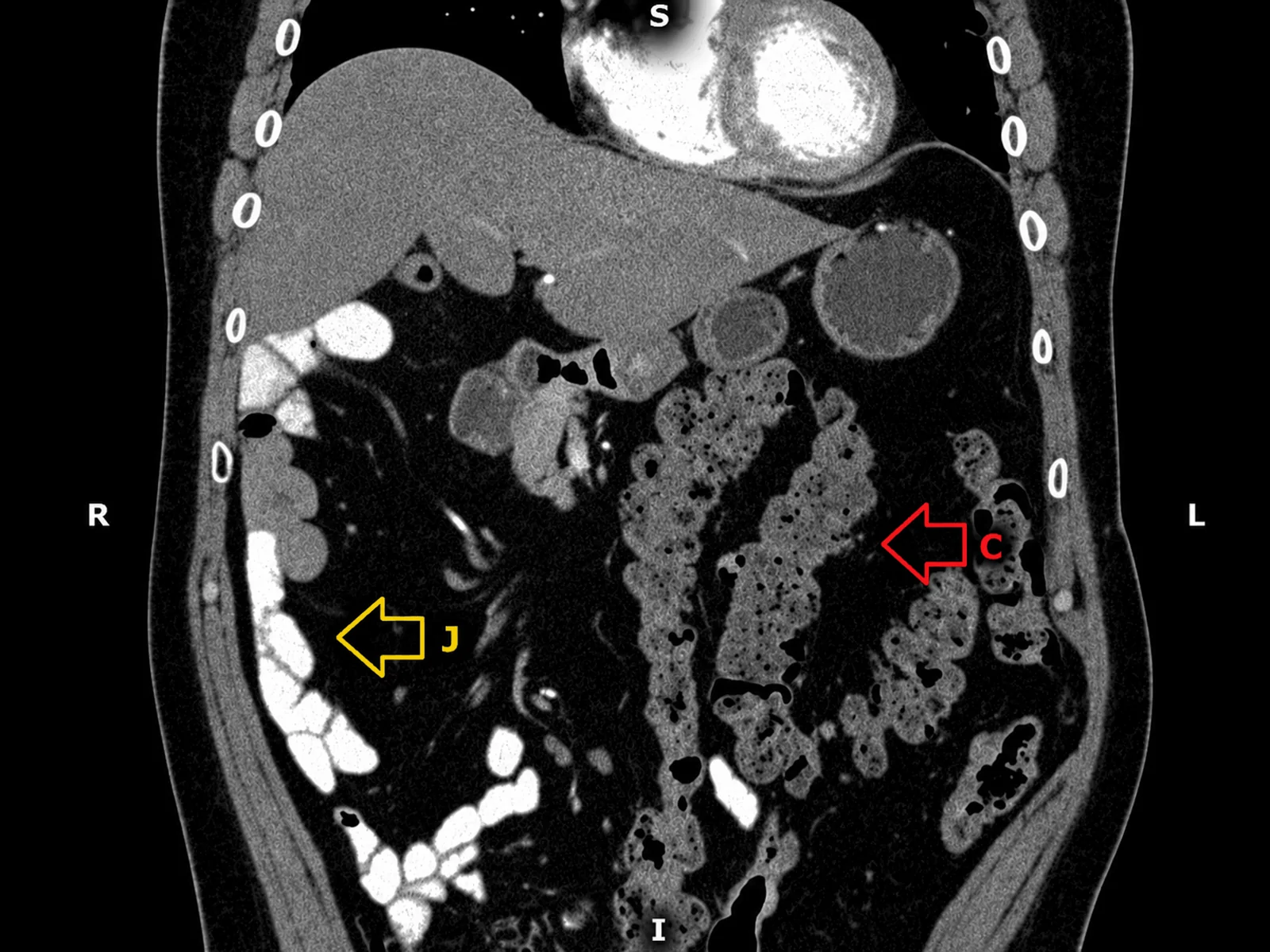
Coronal contrast-enhanced CT image of patient 1 demonstrating right-sided predominance of the duodenum and jejunal loops, a midline course of the ascending colon, and a predominantly left-sided location of the remaining colon. The yellow arrow labeled J indicates the jejunal loops, and the red arrow labeled C indicates the colon. R and L indicate the patient’s right and left sides, respectively, while S and I indicate the superior and inferior directions. CT: computed tomography

**Figure 3:**
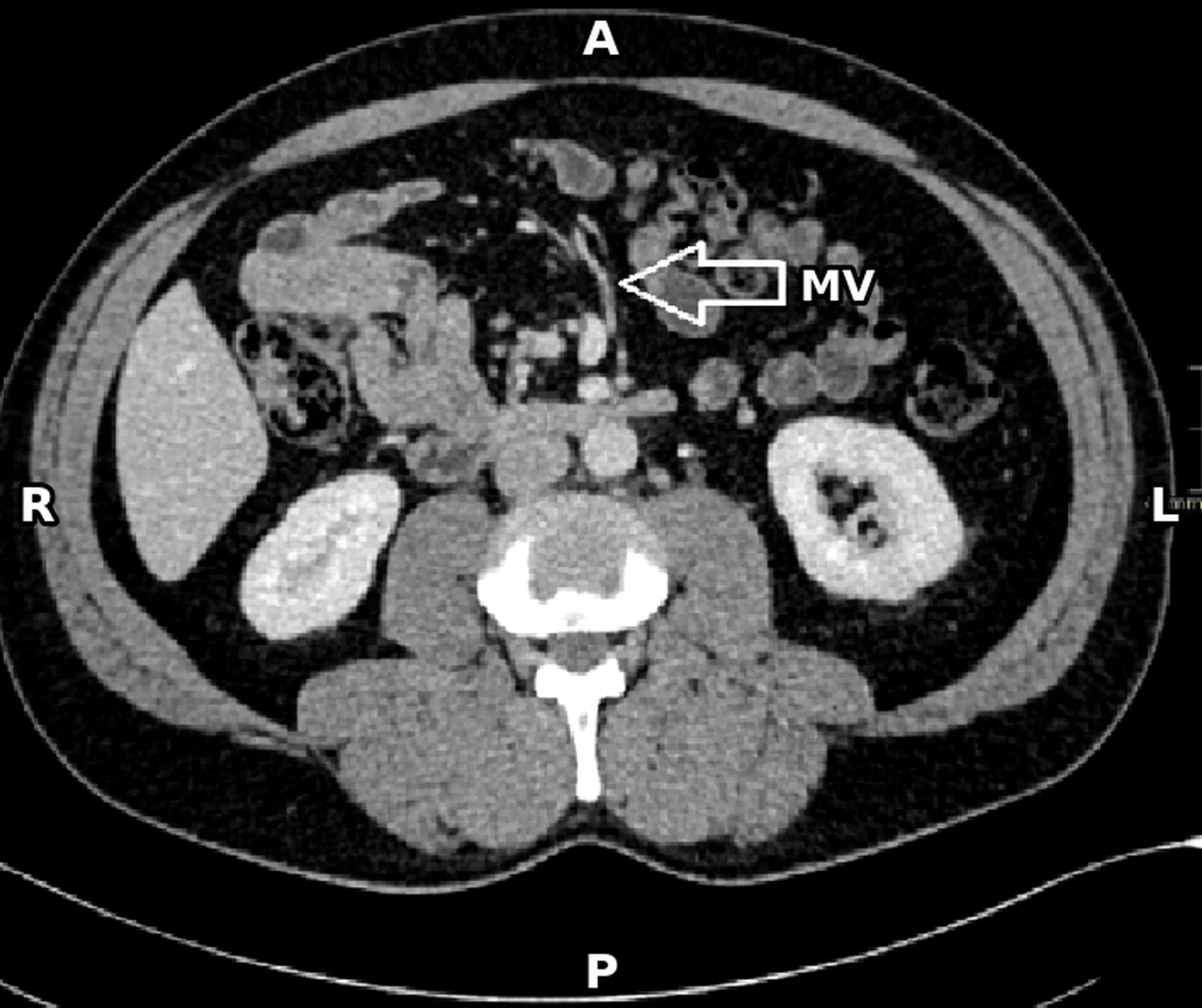
Axial contrast-enhanced CT image of patient 2. The white arrow labeled MV indicates the spiral configuration of the mesenteric vessels. R, L, A, and P indicate the patient’s right, left, anterior, and posterior sides, respectively. CT: computed tomography

**Figure 4:**
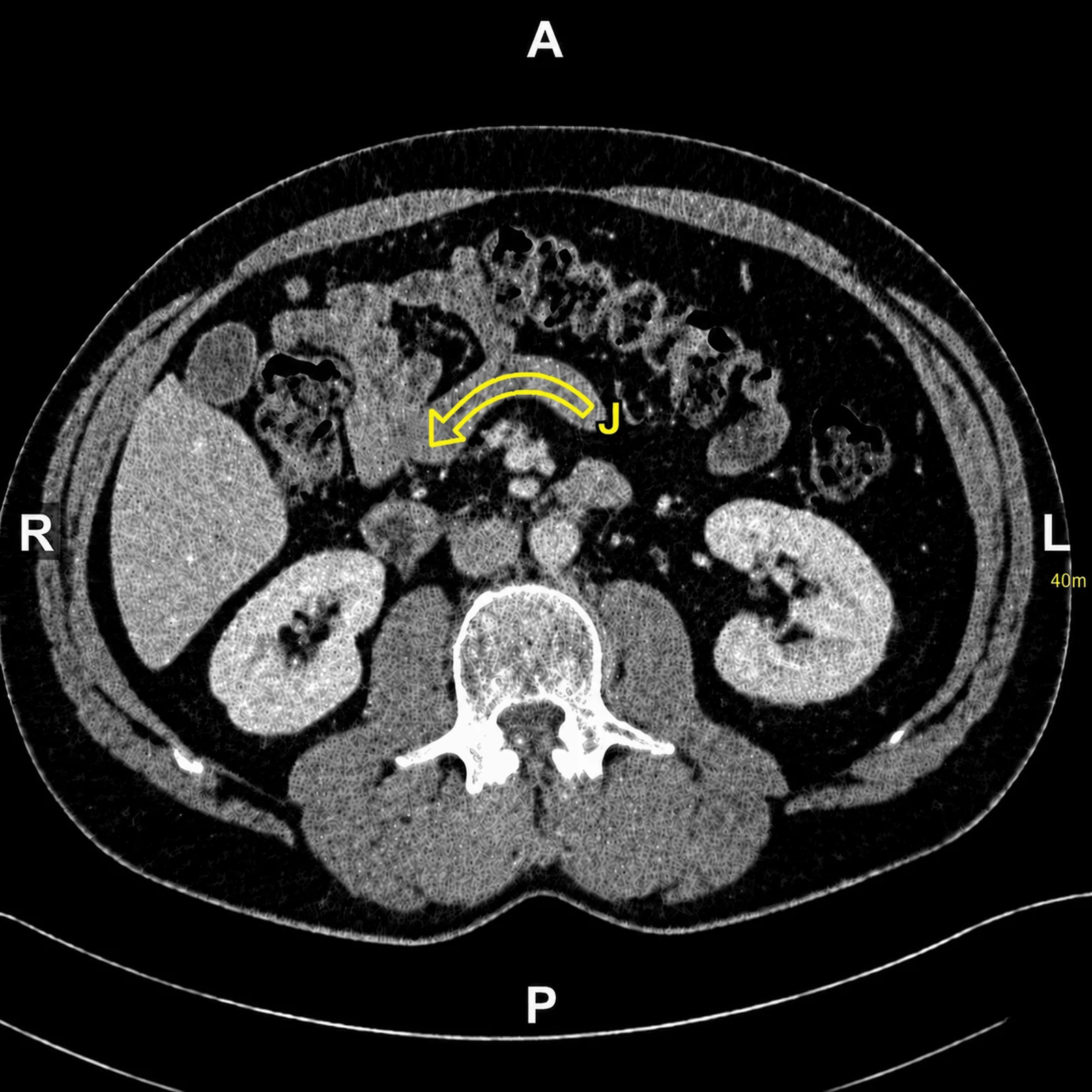
Axial contrast-enhanced CT image of patient 2. The first jejunal loop courses anterior to the superior mesenteric vessels toward the right side, where the remaining small-bowel loops included in the examination are also located, representing features consistent with intestinal malrotation. The colon within the examined region is in a typical anatomical position. The yellow arrow indicates the lumen of the first jejunal loop, which is directed toward the right side. The yellow curved arrow labeled J indicates the lumen and rightward course of the first jejunal loop. R, L, A, and P indicate the patient’s right, left, anterior, and posterior sides, respectively. CT: computed tomography

**Figure 5:**
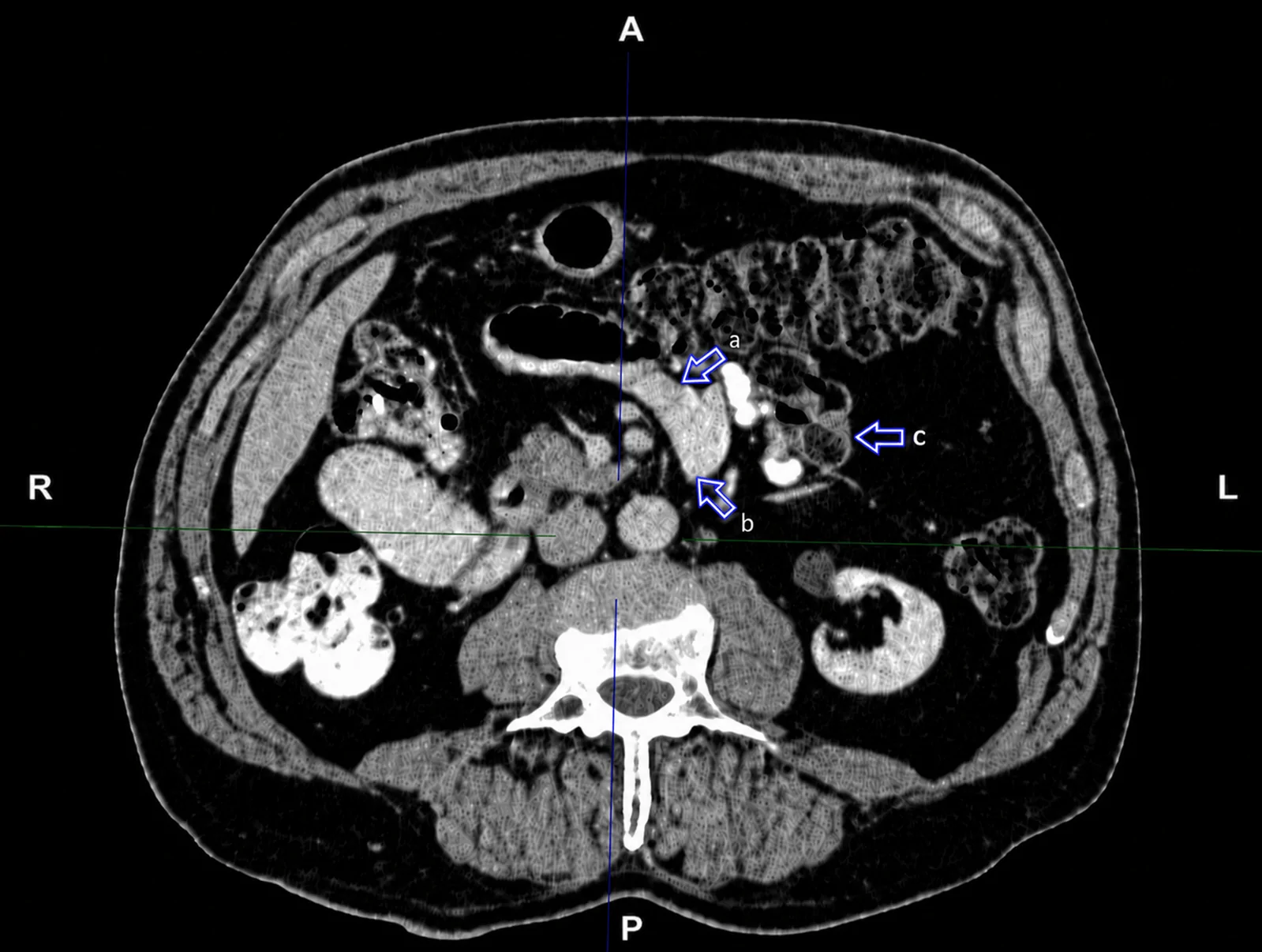
Axial contrast-enhanced CT image of patient 3. Abnormal configuration and course of the duodenum. A small diverticulum is present in the medial part of the bulb, and just below it, there is a relatively narrow passage to the distal part of the duodenum (a), resembling a diverticulum at the site of the passage (b). The lower bend and horizontal part of the duodenum are located low, anterior to the origin of the inferior mesenteric artery from the aorta. The first loop of the jejunum passes to the right side at approximately the level of the pancreas (c). The loops of the small intestine within the scope of the examination are mostly located on the right side, while part of the ileum is located in the left lower abdomen, features consistent with malrotation. R, L, A, and P indicate the patient’s right, left, anterior, and posterior sides, respectively. CT: computed tomography

The identified Xiong subtypes among the seven confirmed cases were DYJRCR (n = 2, 28.6%), DNJRCR (n = 2, 28.6%), DYJRCL (n = 1, 14.3%), DYJRCM (n = 1, 14.3%), and DYJLCR (n = 1, 14.3%). These patterns predominantly involved partial rotation anomalies, allowing for precise anatomical characterization of the identified variants. Representative CT findings are presented in Figures 1-5. Figures 1-2 demonstrate the abnormal right-sided location of the small bowel, the midline course of the ascending colon, and the predominantly left-sided location of the remaining colon in Patient 1. Figures 3-4 show the spiral configuration of the mesenteric vessels and the rightward course of the first jejunal loop in Patient 2. Figure 5 demonstrates the abnormal course of the duodenum and jejunum in Patient 3. Midline crossing of the duodenum and the presence of a retromesenteric D3 segment partially overlapped but did not represent identical findings, reflecting variability in the spatial relationship between the duodenum and the mesenteric vessels.

## Discussion

In this retrospective cohort of adult patients undergoing abdominal CT for diverse clinical indications, intestinal malrotation was confirmed in seven out of 3478 cases (0.20%). These cases fulfilled the predefined CT-based diagnostic criteria; however, the absence of additional imaging or surgical confirmation should be considered when interpreting their diagnostic certainty. This prevalence is consistent with findings from Zissin et al., who showed that incidental rotational anomalies are occasionally detected in asymptomatic adults, but true malrotation remains rare [10]. Importantly, two additional patients in our series demonstrated only atypical bowel positioning, which, after applying strict CT-based criteria, did not meet the definition of malrotation, supporting the observation by Pickhardt and Bhalla, who emphasized that normal anatomic variants are frequently mistaken for malrotation if systematic assessment is not used [1].

Classical radiological signs historically emphasized in pediatric literature showed limited diagnostic utility in our adult cohort. Similar conclusions were reported by Neville et al., who reviewed 194 adult cases of midgut malrotation and found that inversion of the SMA-SMV relationship occurred only in a minority of patients. The authors highlighted that this vascular configuration is highly variable among adults and represents a low-sensitivity and nonspecific marker when used in isolation [4]. Yang et al. identified the absence of a retromesenteric duodenum as the most reliable marker of malrotation [18]. In our cohort, however, five of seven cases demonstrated a preserved retromesenteric D3 course despite abnormalities in jejunal and cecal positioning. This preliminary observation suggests that a preserved retromesenteric D3 segment may not exclude malrotation in all adult patients; however, it should be interpreted cautiously and requires validation in larger studies.

Similarly, in cases where the duodenum did not cross the midline, diagnosis was based on unequivocal deviations in both jejunal and cecal locations, consistent with nonrotation variants. The position of the ligament of Treitz was evaluated as an ancillary sign due to its variability in adults; true malrotation was confirmed based on primary jejunal and cecal displacement.

We observed a distinct distribution pattern: while the jejunum was consistently displaced to the right side, the cecum was frequently found within the RLQ or right abdomen. This highlights that cecal position alone can be misleading in adults. Although the ICV was located in the RLQ in most cases included in Table 2, this finding alone did not indicate normal anatomy, as several confirmed malrotation cases demonstrated a typical RLQ position of the cecum despite clear abnormalities in duodenal and jejunal configuration. Based on the integrated assessment, we assigned each patient to the corresponding Xiong subtype, for example, patterns such as DYJRCR or DYJRCL. Two cases demonstrated only abnormal bowel positioning without fulfilling the definition of malrotation (“atypical bowel positioning” in the Xiong classification), corresponding to the jejunal transposition variants described by the authors. Xiong et al. also reported that 56.6% of their patients were asymptomatic, which aligns with our observation that most individuals in our cohort exhibited minimal or no symptoms [8].

The clinical presentation of malrotation differs markedly between children and adults. In the pediatric population, malrotation typically manifests early in life with bilious vomiting, features of mechanical obstruction, or midgut volvulus, and diagnosis is usually established on the basis of contrast studies and characteristic symptoms. Yang et al. noted that in children, volvulus is the most common complication and often leads to prompt diagnosis [18]. In adults, however, the clinical course is more heterogeneous, most often involving chronic abdominal pain, nonspecific gastrointestinal complaints, or intermittent vomiting. In our series, only one patient presented with complete features of obstruction, consistent with Neville et al., who reported that 87.6% of adults experience at least one chronic symptom, whereas spontaneous volvulus occurs in only 30.9% of cases [4]. However, the available CT-based assessment did not allow this case to be classified as definite acute midgut volvulus. Although a whirlpool-like configuration of mesenteric vessels was observed in two confirmed cases, this finding was interpreted as an ancillary vascular sign rather than proof of surgically confirmed volvulus. This distinction is important because adult malrotation may present across a broad clinical spectrum, ranging from incidental or mildly symptomatic findings to severe obstructive complications. 

Intestinal malrotation in adults is increasingly identified incidentally during abdominal CT performed for unrelated indications. Perez and Pickhardt reported a prevalence of 0.17% among patients undergoing CTC, with a higher rate of 0.75% in individuals with incomplete colonoscopy. Notably, 67% of cases in their study had been previously overlooked, emphasizing the importance of careful assessment of the duodenal course and cecal location even in asymptomatic patients [2]. Similar observations were made by Xiong et al., who noted that most adult patients with malrotation do not exhibit typical symptoms and are diagnosed incidentally [8]. Consistent with these reports, malrotation was an incidental finding in most patients in our cohort. However, the imaging protocol used in our study, routinely including oral and intravenous contrast administration as well as multiphase acquisition, may have facilitated detailed assessment of mesenteric vessels and the duodenal course, potentially increasing the detectability of subtle rotational anomalies. However, the cohort was selected by referral indications requiring abdominal CT, and the routine use of oral and intravenous contrast with multiphase acquisition may have facilitated detailed assessment of mesenteric vessels and the duodenal course. Consequently, the prevalence and imaging characteristics observed in this cohort may not be fully generalizable to unselected adult populations or to populations examined using single-phase or non-contrast CT protocols. This represents a potential source of selection bias that should be considered when interpreting the results. 

This study has several limitations. Its retrospective, single-center design, the inclusion of patients referred for abdominal CT, and the use of a uniform contrast-enhanced protocol limit the generalizability of the findings to unselected adult populations and institutions using different imaging approaches. Complete age and sex data for the entire cohort of 3,478 screened CT examinations were not systematically available in the retrospective dataset, which limited assessment of the representativeness of the screened population. Although the routine administration of oral and intravenous contrast and the predominantly multiphase acquisition enabled detailed assessment of bowel anatomy, mesenteric vessels, and the duodenal course, it may also have increased the detectability of subtle rotational anomalies compared with single-phase or non-contrast CT, introducing potential selection and ascertainment bias.

In addition, diagnoses were based exclusively on CT findings, without additional imaging or surgical confirmation; therefore, subtle or borderline cases may have been misclassified.

Finally, we did not have long-term clinical follow-up for patients with incidentally identified CT findings consistent with intestinal malrotation, which limits our ability to assess the subsequent clinical course of these imaging findings and the potential risk of future complications.

## Conclusions

Intestinal malrotation, although classified as a congenital anomaly of childhood, may remain undiagnosed until adulthood, often presenting with asymptomatic or nonspecific clinical features. Due to its variable clinical presentation and heterogeneous radiological findings, this condition poses a diagnostic challenge and should be considered in the differential diagnosis of both chronic and acute abdominal complaints in adult patients. CT is a pivotal tool for identifying anatomical features suggestive of intestinal malrotation; however, apparent findings on initial imaging do not necessarily correspond to true malrotation. Diagnostic confidence can be improved through systematic application of CT-based criteria assessing the anatomical position of the duodenum, jejunum, and cecum, as individual imaging signs alone are insufficient.

The results of our study indicate that the presence of a single suggestive feature, such as an abnormal SMA-SMV relationship or position of the ICV, cannot be interpreted as conclusive evidence of malrotation, a finding confirmed during the second-stage, comprehensive assessment. However, evaluation of the duodenal course and the location of the jejunum provides valuable guidance in the diagnostic process. The lack of clear guidelines for managing asymptomatic cases and the wide range of clinical presentations necessitate an individualized therapeutic approach, taking into account the risk of potential complications. Further standardization of diagnostic criteria and the development of management recommendations are needed to enable early detection and appropriate treatment of this potentially serious developmental anomaly in adults.

## Figures and Tables

**Figure 6:**
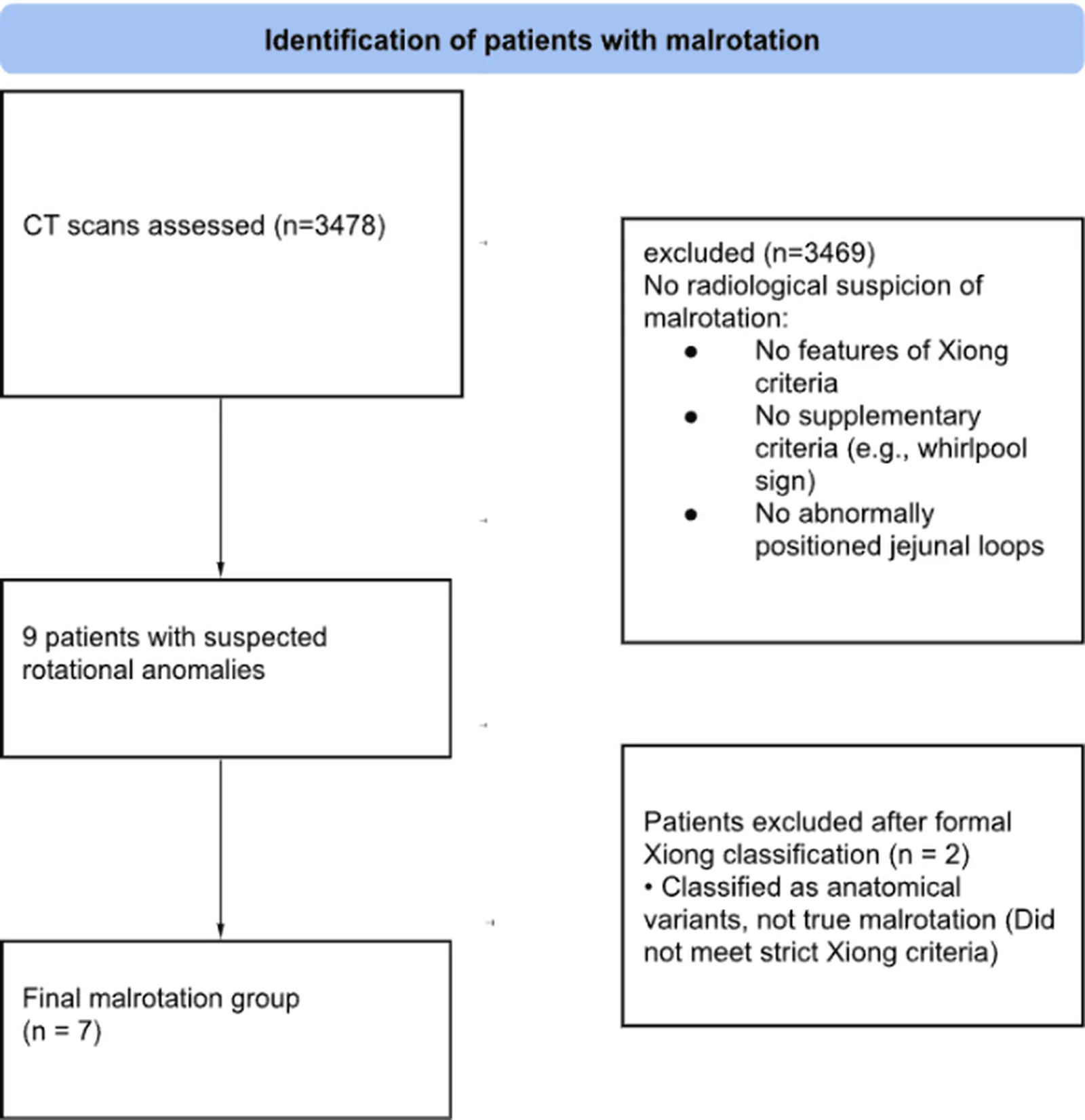
Identification of patients with malrotation.

## Appendices

Appendix 1

**Table 4:** STROBE checklist.

Item No	STROBE Recommendation	Location in Manuscript (Section/Page)
Title and abstract		
1	(a) Indicate the study’s design with a commonly used term in the title or the abstract	Title Page/Abstract (stated as "retrospective cross-sectional study")
	(b) Provide in the abstract an informative and balanced summary of what was done and what was found	Abstract
Introduction		
2	Explain the scientific background and rationale for the investigation being reported	Introduction (Background on malrotation in adults)
3	State specific objectives, including any prespecified hypotheses	Introduction (Last paragraph: objective to evaluate CT criteria)
Methods		
4	Present key elements of study design early in the paper	Methods (Study Design: Retrospective cross-sectional analysis of CT scans)
5	Describe the setting, locations, and relevant dates, including periods of recruitment, exposure, follow-up, and data collection	Methods (Setting: CT studies from June 2023 - April 2025)
6	(a) Give the eligibility criteria, and the sources and methods of selection of participants	Methods (Patient Selection: Inclusion/Exclusion criteria, Xiong criteria)
7	Clearly define all outcomes, exposures, predictors, potential confounders, and effect modifiers. Give diagnostic criteria, if applicable	Methods (Diagnostic criteria: Xiong classification, supplementary signs (duodenal course, jejunal location, SMA-SMV relation, ICV position, whirlpool sign, ligament of Treitz))
8	For each variable of interest, give sources of data and details of methods of assessment (measurement).	Methods (Imaging analysis: Siemens SOMATOM definition)
9	Describe any efforts to address potential sources of bias	Methods (Bias control: Independent assessment by two blinded radiologists Z.M. and J.K.; a third expert adjudicator resolved disagreements). Interobserver agreement reported in Results.
10	Explain how the study size was arrived at	Methods / Results (Analysis of all 3478 consecutive scans available in the study period)
11	Explain how quantitative variables were handled in the analyses.	Methods (Statistical Analysis section)
12	(a) Describe all statistical methods, including those used to control for confounding	Methods (Statistical Analysis: Wilson score interval, Fisher exact test)
	(b) Describe any methods used to examine subgroups and interactions	N/A (Exploratory study)
	(c) Explain how missing data were addressed	Results (No missing data reported for included cases)
Results		
13	(a) Report numbers of individuals at each stage of study	Results (Flow: 3478 CT → 9 suspected → 7 confirmed; screening process described)
	(b) Give reasons for non-participation at each stage	Results (Exclusion of positional variants)
	(c) Consider use of a flow diagram	See Figure 6 (Flow Diagram)
14	(a) Give characteristics of study participants (eg demographic, clinical, social)	Results (Demographics, clinical presentations, and malrotation subtypes described in text and tables)
15	Report numbers of outcome events or summary measures	Results (Demographics, clinical presentations, and malrotation subtypes described in text and tables)
16	(a) Give unadjusted estimates and their precision (eg, 95% confidence interval).	Results (Prevalence: 0.20% (95% CI); Symptoms: 28.6% (95% CI))
17	Report other analyses done - eg analyses of subgroups and interactions, and sensitivity analyses	Results (Clinical correlation section)
Discussion		
18	Summarise key results with reference to study objectives	Discussion (First paragraph)
19	Discuss limitations of the study, taking into account sources of potential bias or imprecision.	Discussion (Limitations paragraph: Small sample size N=7, retrospective, single-center design, clinical symptoms extracted retrospectively from referral forms)
20	Give a cautious overall interpretation of results considering objectives, limitations, multiplicity of analyses	Discussion (Conclusion regarding exploratory nature, malrotation in adults is rare and usually asymptomatic, an abnormal duodenal course is the most reliable diagnostic marker)
21	Discuss the generalisability (external validity) of the study results	Discussion (Based only on CT images, no long-term observation was conducted)
Other information		
22	Give the source of funding and the role of the funders for the present study	Declarations/Funding (e.g., None declared)

Appendix 2
